# Label-Free, Flow-Imaging Methods for Determination of Cell Concentration and Viability

**DOI:** 10.1007/s11095-018-2422-5

**Published:** 2018-05-30

**Authors:** A. S. Sediq, R. Klem, M. R. Nejadnik, P. Meij, Wim Jiskoot

**Affiliations:** 1 Division of BioTherapeutics, Leiden Academic Centre for Drug Research (LACDR), Leiden University, Leiden, The Netherlands; 2 Department of Clinical Pharmacy and Toxicology, Leiden University Medical Center, Leiden, The Netherlands

**Keywords:** cell based medicinal product, cell viability, FlowCAM, hemocytometry, micro-flow imaging, particle analysis

## Abstract

**Purpose:**

To investigate the potential of two flow imaging microscopy (FIM) techniques (Micro-Flow Imaging (MFI) and FlowCAM) to determine total cell concentration and cell viability.

**Methods:**

B-lineage acute lymphoblastic leukemia (B-ALL) cells of 2 different donors were exposed to ambient conditions. Samples were taken at different days and measured with MFI, FlowCAM, hemocytometry and automated cell counting. Dead and live cells from a fresh B-ALL cell suspension were fractionated by flow cytometry in order to derive software filters based on morphological parameters of separate cell populations with MFI and FlowCAM. The filter sets were used to assess cell viability in the measured samples.

**Results:**

All techniques gave fairly similar cell concentration values over the whole incubation period. MFI showed to be superior with respect to precision, whereas FlowCAM provided particle images with a higher resolution. Moreover, both FIM methods were able to provide similar results for cell viability as the conventional methods (hemocytometry and automated cell counting).

**Conclusion:**

FIM-based methods may be advantageous over conventional cell methods for determining total cell concentration and cell viability, as FIM measures much larger sample volumes, does not require labeling, is less laborious and provides images of individual cells.

**Electronic supplementary material:**

The online version of this article (10.1007/s11095-018-2422-5) contains supplementary material, which is available to authorized users.

## Introduction

Cell-based medicinal products (CBMPs) are receiving increasing attention by the pharmaceutical industry because of their potential in treatment of a variety of diseases, such as cancers, viral infections, and autoimmune disorders (1,2). Like for any other pharmaceutical drug product, the quality of CBMPs highly determines their safety and efficacy (3).

The safety of a CBMP depends, amongst others, on the presence of cellular and non-cellular impurities. When a specific cell type is required for the therapy, unwanted cell populations are considered impurities and should be tested and controlled. Other impurities can be related to the process of manufacturing, for instance, traces of raw materials used, like micro carrier beads.

Efficacy of the CBMPs is in mainly determined by cell-specific functional properties; however the cell viability and total concentration of the cells is also important in this. During production, starting cell materials undergo different manipulations, such as harvesting, purification, genetic manipulation, expansion, freezing and thawing (4). These process steps, as well as other manipulations, such as storage, transport (5,6), and preparation for administration to the patient (7), can induce different types of stresses to the cells and potentially affect cell viability. Therefore, viability and total cell concentration are considered important quality attributes and should be measured as in-process control, at release and during stability testing.

There are several methods to determine cell viability and they all have their pros and cons. Despite continuous development of new methods for assessing cell viability, hemocytometry (8), automatic cell counting and flow cytometry (9) are still the most frequently applied techniques for this purpose. The flow cytometer provides information about cell size and granularity, respectively. Granularity level and size has been shown to be inversely related with cell viability (10). The use of fluorescent dyes or fluorescently labeled antibodies directed against cell surface markers can aid in the evaluation of cell type and viability. Flow cytometry is considered to be a very accurate and reproducible technique for cell viability tests, but is also considered very laborious. The sample preparation including, e.g., labeling of the cells, can be time-consuming and expensive, and validation of flow cytometric methods has been reported to be challenging (11).

The determination of cell viability with a hemocytometer is based on staining of the dead cells using dyes like eosin and trypan blue (8). Hemocytometry is currently the gold standard in clinical practice for cell counting and viability determination. Whilst being a fast method, the method can be laborious and has certain weak points (e.g. errors originating from sample preparation; low analysis volume (0.1 μL)) that potentially compromise the accuracy of the method. With respect to the quantity of cell suspension measured and the laborious nature of the method, automated cell counters could provide improvement, especially for routine measurements, however the analysis volume is still low (0.4 μL). Despite the higher throughput of the automated cell counter compared to the hemocytometer (12).

In this regard, less laborious, inexpensive techniques that allow for rapid and reliable counting of CBMPs would be beneficial for improving the quality and success of CBMPs in clinical practice. Emerging flow imaging microscopy (FIM) techniques may fulfill these needs. In these systems the sample flows through a flow cell where images are taken with a high-magnification digital camera. With the help of the dedicated instrument software, the quantity and several morphological parameters of the particles can be extracted from the images (13). FIM can give valuable information about cells, without the need for labeling, and detect small changes in cell size and morphology which have been shown to be related to cell viability (14,15). In addition, FIM techniques are generally easy and fast to perform.

One of these FIM techniques is Micro-Flow Imaging (MFI), which owes it popularity in this field mainly to its user-friendliness and robust operation. The application of MFI for cells is limited but not unexplored. For instance, Martin *et al*. have used MFI to study aggregation tendency of thawed hematopoietic stem cells (16). A recent study of Farrell and coworkers used MFI to determine cell confluency on micro-carriers used in culture-derived bioreactors (17). Besides MFI, FlowCAM has been explored for its potential in drug product development (18–20) and drug delivery systems (21). Even though MFI and FlowCAM are based on the same measurement principle, they differ in several aspects. The FlowCAM has a higher resolution and provides more particle parameters, whereas the MFI tends to provide a more accurate determination of particle concentration (20,22).

The aim of this study was to evaluate the feasibility of two FIM techniques, i.e., MFI and FlowCAM, to determine the total cell concentration and cell viability. In the presented study, the two techniques are used for the characterization of two cell lines, stored for up to 8 days under ambient conditions, and compared with hemocytometry and automated cell counting as well as with each other.

## Materials and Methods

### Cell Materials

B-lineage acute lymphoblastic leukemia (B-ALL) cells were used as model cells in this study. The cells were cultured from two different donors (ALL-CR and ALL-CM (23); further referred to as cell line 1 and cell line 2) and provided by the Department of Hematology, Leiden University Medical Center (LUMC, Leiden, the Netherlands). Cells were frozen in 60% *v*/v wash medium (BioWhittaker® Iscove’s Modified Dulbecco’s Medium (IMDM) 98.5% *v*/v, penicillin/streptomycin (Lonza) 1% v/v, HSA-20% 0.5% *v*/v) and a final concentration of 10% v/v HSA-20%, 10% v/v DMSO (LUMC, Leiden, the Netherlands) at a concentration of about 1 × 10^7^ cells/mL and stored at −80°C until the start of incubation experiments. After controlled thawing, washing and counting (using a hemocytometer), cells were suspended in NaCl 0.9% m/v + HSA-20% 2% *v*/v at a concentration of 10^6^ total cells/mL and were exposed to the stress condition described below.

### Stress Condition

The chosen stress condition was storage at ambient conditions (on the lab bench) for up to 8 days, during which the cells showed a gradual and reproducible decrease in viability, as demonstrated in a pilot experiment. The total cell concentration and morphological parameters (see below) of each sample were analyzed at different days by using MFI and FlowCAM. For cell line 1 the measurements were performed on day 0, 1, 2, 4, 6 and 8. The study performed with the 2nd cell line served as a confirmative study and therefore the measurements on day 4 and 6 were not performed. In parallel, the total cell concentration and number of viable cells in the same samples were determined with a hemocytometer and an automated cell counter. For the data analysis of both flow imaging microscopy techniques we only included particles with a size ≥4 μm, which is the lowest detectable particle size for the automated cell counter, according to the manufacturer.

### Fluorescence-Activated Cell Sorting (FACS)

Fluorescence-activated cell sorting (FACS) on a FACSAria III (BD Biosciences, New Jersey, USA) was used to separate dead and/or dying cells from living cells based on the forward scatter (FSC) and side scatter (SSC) ‘live gate’ as shown in Supplementary Fig. S1. The presumably dead and live cells that were collected with the FACS were then measured with MFI and FlowCAM. These FIM data were used to develop software filters for dead and live cells for each FIM technique (see results section).

### Hemocytometry

Cell suspensions were diluted twofold with a trypan blue solution (0.4% (*w*/*v*) in 0.81% m/v NaCl and 0.06% m/v K_2_HPO_4_ (Bio-Rad, Hercules, California, USA)). Ten μL of the mixture was placed on a Bright-Line hemocytometer glass (Sigma-Aldrich, Steinheim, Germany), and analyzed by using a light microscope (Zeiss Axiostar Plus, Carl Zeiss Light Microscopy, Göttingen, Germany) with 10× magnification. Both viable (not stained) and non-viable (stained) cells were counted in 25 frames of the hemocytometer according to the manufacturer’s recommendations and the percentage of viable cells to the total was calculated (24). For each sample triplicate measurements were conducted.

### Automated Cell Counting

Ten μL of the mixture prepared for the hemocytometry was introduced into the counting slide. Subsequently, the total cell concentration and percentage of viable cells were measured by using a Bio-Rad TC20 Automated Cell Counter (Bio Rad, Hercules, California, USA). This procedure was repeated three times for each sample.

### Micro-Flow Imaging (MFI)

In order to reduce the probability of detection of optically overlapping particles, cell-containing samples were first diluted 4-fold with particle free NaCl 0.9% m/v + HSA-20% 2% *v*/v. The diluted samples were analyzed by using a Micro-Flow Imaging 5200 (Protein Simple, Santa Clara, CA, USA), with MFI View System Software (MVSS) Version 2. No software filters were applied during the runs. The 100-μm silane coated flow cell was rinsed with flow of ultrapure water (18.2 MΩ.cm; dispensed by using a Purelab Ultra water purification system, ELGA LabWater, Marlow, UK) and thereafter a background measurement was taken with particle free NaCl 0.9% m/v + HSA-20% 2% *v*/v. For the analysis, 0.50 ml of each sample was run at a flow rate of 0.17 mL/min. The data analysis was performed with MFI View Analysis Suite (MVAS) Version 1.2. For the data analysis, the lower size limit was set at 2 μm in order to avoid analysis on the edge of the detectable particle size range (i.e.*,* 1 μm). The upper size limit was set at 20 μm because particles larger than that were most likely contaminants (e.g., dust) and contributed to less than 0.1% of the total particle concentration. Table I summarizes the main morphological parameters provided by the MVAS and their descriptions. The size distribution of each sample was presented in equivalent circular diameter (ECD). Each sample was measured three times with MFI.

**Table I:** Morphological parameters used in this study and their descriptions as provided by MVAS (MFI) and Visual SpreadSheet (FlowCAM)

Parameter	Unit	Description
Micro-Flow Imaging		
Equivalent circular diameter (ECD)	Microns	The diameter of a circle occupying the same area as the particle
Intensity mean	Intensity (0–1023)	The average intensity of all image pixels representing the particle
Intensity standard Deviation	Intensity (0–1023)	The standard deviation of the intensity of all pixels representing the particle
Circularity	No units (0–1)	The circumference of a circle with an equivalent area divided by the actual perimeter of the particle
Aspect ratio	No units (0–1)	The ratio of the minor axis length over the major axis length of an ellipse that has the same second-moment-area as the particle
FlowCAM		
Area based diameter (ABD)	Microns	The diameter based on a circle with an area that is equal to that of the particle
Equivalent spherical diameter (ESD)	Microns	The mean value of 36 feret measurements (the perpendicular distance between parallel tangents touching opposite sides of the particle; VisualSpreadsheet makes 36 feret measurements for each particle, one each 5 degrees between −90 degrees and + 90 degrees)
Symmetry	No units (0–1)	A measure of the symmetry of the particle around its center; if a particle is symmetric, then the value is one
Aspect ratio	No units (0–1)	The ratio of the width (the shortest axis of the particle) and length (the longest axis of the particle)
Circle fit	No units (0–1)	Deviation of the particle edge from a best-fit circle, normalized to the zero to one range where a perfect fit has a value of one
Circularity	No units (0–1)	A shape parameter computed from the perimeter and the area; a circle has a value of one (formula: (4 x π x Area) / Perimeter^2^)

### FlowCAM

The second flow imaging technique used in this study was a FlowCAM VS1 (Fluid Imaging Technologies, Yarmouth, ME, USA). After rinsing the FC50 flow cell with ultrapure water, 100 μL of each 4-fold diluted sample was run at a flow rate of 0.030 ml/min controlled by a C70 syringe pump. Images were taken with a Sony XCD-SX90 camera at 22 fps (shutter: 8, gain: 224, 20× lens). The data were analyzed by Visual SpreadSheet Version 3. For reasons described in the MFI section, only particles between 2 and 20 μm were included in the data analysis. In order to remove edge particles (particles that were detected at the borders of the camera field, hence imaged partially), the acceptable detection field was reduced to 95–1183 and 6–952, respectively, for left-right and top-bottom orientations. The edge gradient parameter provided by FlowCAM was used to exclude out-of-focus particles. The acceptable range for edge gradient was determined in a preliminary study. In Table I, descriptions of the main morphological parameters provided by the Visual SpreadSheet are given. It is worth mentioning that the FlowCAM can calculate the particle size through two different algorithms (described in Table I). In our study we chose to proceed with the area based diameter (ABD), because the principles of ABD and ECD are similar.

### Definition of Software Filters to Discriminate Live and Dead Cell Populations

Based on measurements of the FACS-sorted live and dead particle poplulations (see above), the parameter that showed the largest difference (ECD for MFI and ABD for FlowCAM) between live and dead cells, was used as a primary filter. After applying this primary filter, the changes of all the other parameters were evaluated and their threshold values could systematically be fine-tuned. At the end, the established set of morphological filters was tested on the analyzed sorted fractions and FIM-derived viability was compared to the trypan blue-assisted values found for each cell sample (see results section for details).

## Results

The two B-ALL cell lines were thawed, analyzed and then left at ambient temperature for 8 days and analyzed at predetermined time points, as described in materials and methods.

### Monitoring Total Cell Concentration Over the Eight-Days Study Period

**Fig. 1:**
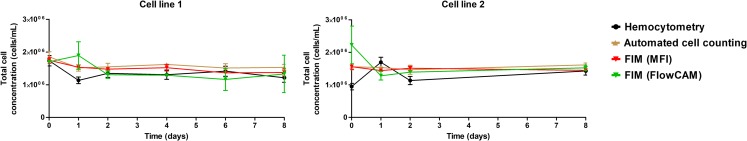
Total cell concentration of (**a**) cell line 1 and (**b**) cell line 2 during 8 days of storage at ambient conditions measured by hemocytometry (black), automated cell counting (red), MFI (yellow) and FlowCAM (blue). Error bars represent the standard deviation of triplicate measurements with each technique.

Figure 1 shows the total (live and dead) cell concentrations as measured with all four techniques over the 8-days study period, for both investigated cell lines. The results indicate that all the techniques gave fairly similar total cell concentrations. FlowCAM appeared to have the lowest precision, followed by hemocytometry, as judged by the standard deviations. The total cell concentration of cell line 1 showed a slowly decreasing trend over time, whereas for cell line 2 the cell counts remained fairly stable.

### Sorting and FIM Derived Morphology of Dead and Live Cells

The fresh cell suspension of cell line 1 was analyzed with a flow cytometer to derive an appropriate gate for sorting dead and live cells (see Supplementary Fig. S1). After the fractions were collected, a control trypan blue assisted viability test of each fraction was performed on the automated cell counter. From these control measurements, it was found that the live population contained almost 90% viable cells, whereas the dead population contained no more than 20% viable cells. For comparison, the viability of the unfractionated and unstressed cell population was about 75%.

**Fig. 2:**
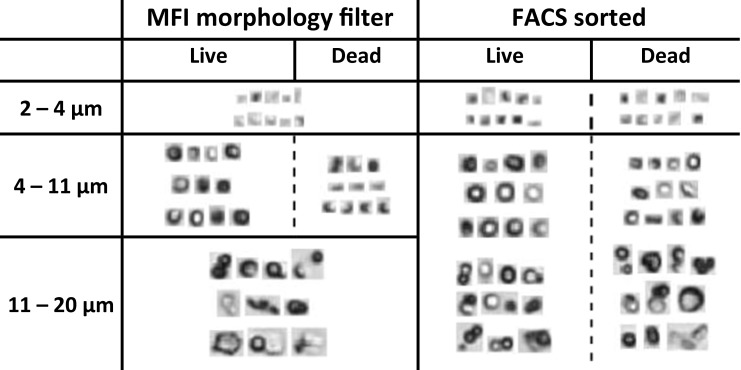
Representative images of particles detected by MFI in a B-ALL cell population. The left column shows the particles that were identified as dead or live cells based on the morphological filters. The right column shows images of particles that were found in FACS-assisted sorted samples of live and dead cells. The MFI morphological filter (for dead and live cells) uses only the size in the range between 4 and 11 μm.

**Fig. 3:**
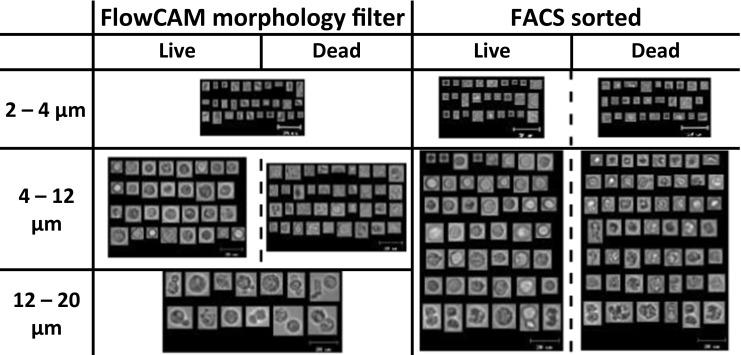
Representative images of particles detected by FlowCAM in a B-ALL cell population. The left column shows the particles that were identified as dead or live cells based on the morphological filters. The right column shows images of particles that were found in FACS-assisted sorted samples of live and dead cells. The FlowCAM morphological filter (for dead and live cells) uses only the size in the range between 4 and 12 μm.

The sorted populations were measured with both FIM techniques. Although it was difficult to see the differences visually (Figs. 2 and 3), evaluation of different morphological parameters for live and dead cells showed a statistically significant difference between the values of each listed parameter for live and dead cells, except for the circularity values derived from MFI and FlowCAM (Table II). The filters were constructed by setting one value at a time starting from the most distinctive parameter until no change was observed in the populations. Using this approach we defined software filters based on the monitored morphological parameters for dead and live cells, as shown in Table III.

**Table II:** Derived Morphological Parameters (mean **±** standard deviation) Provided by MFI and FlowCAM for the Two Cell Fractions of Cell Line 1 Sorted Using FACS

Flow imaging microscopy morphological parameters*	Live cell population	Dead cell population	R^2^*
Micro-Flow Imaging			
ECD	7.6 ± 2.2 μm	5.8 ± 1.8 μm	0.171
IntMean	546 ± 87	573 ± 81	0.026
IntSD	179 ± 53	173 ± 55	0.003
Cir	0.88 ± 0.06	0.88 ± 0.05	0
AR	0.85 ± 0.12	0.87 ± 0.10	0.008
FlowCAM			
ABD	7.7 ± 3.0 μm	6.4 ± 2.6 μm	0.055
Sym	0.74 ± 0.19	0.69 ± 0.17	0.019
AR	0.82 ± 0.16	0.80 ± 0.13	0.005
CF	0.77 ± 0.17	0.75 ± 0.13	0.004
Cir	0.78 ± 0.17	0.78 ± 0.12	0

*Statistical comparison of the morphological parameters of the dead and live cells. The comparison is derived after applying t-test with GraphPad Prism 5®. R^2^ quantifies the fraction of all the variations in the samples that is accounted for by a difference between the group means

**Table III:** Specification of the Software Based Morphological Filters Used to Identify Dead And Live Cells from Analysis Results of the FIM Methods

Flow imaging microscopy morphological parameters	Filter for live cell population	Filter for dead cell population
Micro-Flow Imaging		
ECD	7.25–11 μm	4–7.25 μm
IntMean	≤ 550	≥ 550
IntSD	≤ 170	≥ 170
FlowCAM		
ABD	7–11 μm	4–9 μm
Sym	≥ 0.7	≤ 0.8
AR	≥ 0.8	0.6–0.8
CF	≥ 0.7	≤ 0.8
Cir	≥ 0.8	0.3–0.8

### Comparing Cell Viability Determination by FIM Techniques, Hemocytometry and Automated Cell Counting

**Fig. 4:**
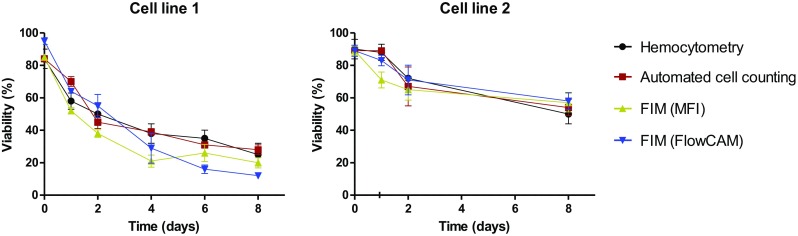
Cell viability determined with different analytical methods for cell line 1 at different time points during 8 days of storage at ambient conditions: hemocytometry (black), automated cell counting (red), MFI (yellow) and FlowCAM (blue). The error bars represent standard deviations of triplicate measurements with each method.

With the help of the established software filters, the percentage of viable cells, i.e., cell viability, was calculated for both cell lines at different storage time points (Fig. 4). These percentages obtained from different techniques showed similar trends for the viability of both cell lines, i.e., a gradually decreasing viability over incubation time. In addition, cell line 2 showed a stronger survival at the studied incubation conditions (75% decrease in viability after 8 days) than cell line 1 (50% decrease).

### Morphological Parameters Obtained by FIM Techniques

Both FIM techniques provide morphological parameters of the detected particles (including cells) obtained from the individual images. Representative images of individual particles detected by the two FIM techniques are shown in Figs. 2 and 3. When comparing these cell images derived from MFI and FlowCAM, it is obvious that FlowCAM has a much higher lens magnification. The high-resolution images of FlowCAM may result in the ability to derive more morphological parameters as compared to MFI. Nevertheless, some of the parameters allowed a comparison of FlowCAM with MFI. In our 8-days study we monitored changes in all the 5 parameters listed in Table I for the studied cells. Most of these parameters showed a trend of a gradual change in the course of the 8-day study.

**Fig. 5:**
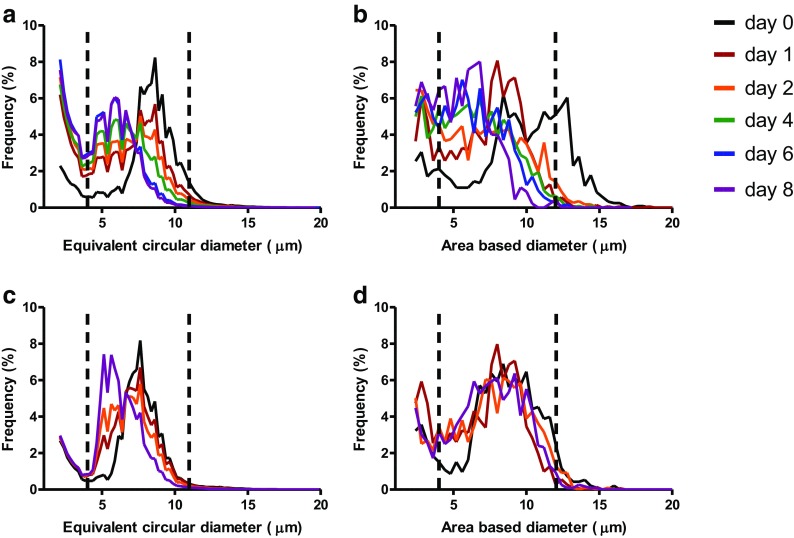
Size frequency distribution of the particles encountered in samples of cell line 1 (**a**, **b**) and cell line 2 (**c**, **d**) measured with MFI (**a**, **c**) and FlowCAM (**c**, **d**) during 8 days of storage at ambient conditions. The size range actually used for the determination of total cell concentration and cell viability by using morphological filters is indicated with the vertical dotted lines.

Figure 5 shows the size distribution of particles derived from the MFI and FlowCAM analysis for both cell lines during the 8-days incubation study. All the size distribution graphs show that there was a decrease in the number of the larger particles and an increase in the number of smaller particles over time. When focusing on cell line 1 (Fig. 5a, b), it is obvious that the peak around 8 μm in the MFI-derived distribution on day 0 slowly descended, while a new peak around 6 μm arose and became apparent on day 8. For FlowCAM, the change in the ABD size distribution was fairly similar to the changes seen in the ECD size distribution with MFI. Fresh cell sample showed a distinct peak at about 12 μm and over time this peak disappeared and was replaced by relatively broad peak at a smaller size range (around 6 μm). However, there was also a third transient peak at about 8 μm seen, which was already present in the fresh sample and increased after 24 h, and then diminished during the following days. MFI and FlowCAM also showed similar trends for cell line 2, which however showed a somewhat smaller cell size for the main population on day 0.

For the determination of viability only the size range between 4 and 11 μm and 4–12 μm (indicated by the dotted vertical lines in Fig. 5) were used with MFI and FlowCAM, respectively. The lower size limit was fixed for both methods, because the lowest detectable particle size of the automated cell counter is 4 μm. Particles below 4 μm may consist of fragmented cells or cell debris. Particles above 11 or 12 μm include clustered cells or aggregates. From the size distribution graphs in Fig. 5 it is seen that the concentrations of presumably cell debris (particle size 2–4 μm) and clustered cells or aggregates (particle size 11–20 μm for MFI and 12–20 μm for FlowCAM) change over time. The concentration of the 2–4 μm particles steadily increased as measured with MFI. With FlowCAM the increase was only apparent during the first few days. With respect to the aggregates, both FIM techniques showed decreasing concentrations.

Changes were also observed there during the 8-day study in the other morphological parameters of both FIM techniques. The details are shown in the Supplementary Information (see Supplementary Fig. S2-S5 for both cell lines).

## Discussion

Viability and total cell concentrations are important quality attributes of CBMPs. The availability of a rapid, easy and reliable method is highly beneficial for viability measurement of cells from starting material procurement throughout the drug product manufacturing process including CBMP release testing, storage, shipment and administration to the patient.

In this study we have investigated the potential of two different FIM techniques to assess the total cell concentration and cell viability. For this purpose, the cells were placed at ambient conditions for up to 8 days, because pilot studies had shown that this is a convenient way to reproducibly obtain changes in viability as function of time. Moreover, the chosen conditions are relevant from a clinical perspective, as cells are commonly kept for some time at room temperature before administration. Our data show that both techniques can be used to measure the concentration and the viability of cells, yielding comparable results to those obtained with conventional cell counting methods.

The FIM methods, once developed, are easy to perform and do not require labeling of the cells before the measurement (see comparison made in Table IV). These techniques measure a relatively large sample volume (of 4-fold diluted sample) and thus count considerably more cells than the conventional methods such as hemocytometry. This ability is a great asset for the accuracy and precision of both total cell concentration and cell viability determinations. Another advantage of FIM lies in its ability to image individual cells and obtain morphological characteristics of each detected cell. Moreover, non-cellular materials (e.g., beads) can be manually removed from the data, to avoid inaccurate counting. Furthermore, the imaging capability of especially FlowCAM may allow the discrimination of different types of cells in a heterogeneous cell population.

**Table IV:** Comparison of the Characteristics of the Techniques Evaluated in this Study

Characteristics	Hemocytometry	Automated cell counting	FIM (MFI)	FIM (FlowCAM)
General				
Analyzed sample volume	0.1 μL	0.4 μL	260 μL	20 μL
Sample pretreatment	Labeling; dilution if needed	Labeling; dilution if needed	Dilution	Dilution
Analysis time per sample (measurement + data analysis)	5 min	1 min	15 min	10 min (depends on measurement settings)
Cell counting and viability determination				
Accuracy*	Moderate	Moderate	High	Moderate
Precision^$^	Moderate	Moderate	High	Low
Additional features				
Non-cellular particles	Discarded visually from the cell counts	May interfere with the cell counting	Can be removed from the data afterwards	Can be removed from the data afterwards
Detection of cell debris	Depends on magnification lens used	Not possible	Possible	Possible
Cell identification	Visual identification	Not possible	Probable, when using morphology based software filters	Highly probable, when using morphology based software filters

*Determined based on the effectively measured sample volume, extrapolation factor to final cell concentration unit (e.g., cells/mL)

^$^Determined based on the outcome of our study

Although hemocytometry, automated cell counting, MFI and FlowCAM are all able to provide an estimation of the total and viable cell concentration, standard deviations presented in Fig. 1 indicate that the precision of methods with respect to the cell concentration measurement follows this pattern: MFI > automated cell counter > hemocytometry > FlowCAM. For FlowCAM, the combination of the type of flow cell and image frequencies used in our settings resulted in a theoretical analysis efficiency of around 20%, meaning that only 20% of the dispensed cell suspension was actually imaged. This limitation in combination with the inability of the analysis package in exclusion of stuck particles (i.e., particles adhered to the measurement cell) that appear in several images from the analysis may be the most important contributing factors to the relatively low precision. Despite the larger amount of suspension volume measured with the automated cell counter (cf. Table IV), automated cell counting did not offer a much better precision over hemocytometry with respect to total cell concentration. This may be caused by the interference of non-cellular material (e.g., contaminants such as dust) with the cell counting. In contrast, MFI resulted in the highest precision. The relatively large volume of imaged sample (more than 65 μL *vs*. 0.1 μL in hemocytometry), high analysis efficiency (about 85%) and ability to remove stuck particles (see above) may explain the pronounced performance of MFI with respect to the total cell concentration determinations. Moreover, the precision of hemocytometry may be affected by the operator, since the method requires visual counting and viability assessment based on visual discrimination of the color of the cells.

Stability studies mimicking the clinical conditions of cell preparation for administration and storage at ambient conditions (i.e., in-use stability) are very important to establish whether the cells are still viable upon administration. Monitoring of the total cell concentration of cell line 1 over storage time revealed a decreasing trend in total cell concentration for all the methods. It is known that dying and dead cells undergo fragmentation into smaller particles (25). These cell debris particles are below the lower size limit of the automated cell counting (4 μm) and the lower size limit chosen for FIM techniques, while in hemocytometry non-cellular particles are visually excluded and therefore not counted. FIM data confirm this, as the ECD and ABD size distribution diagrams (Fig. 5) show a clear increase in the number of particles below 4 μm over time, especially within the first 24 h of incubation. This observation suggests that the FIM techniques are presumably able to detect and count cell fragments as well, which may be useful for product characterization. However, it is not easy to reliably determine morphological parameters of these small particles, which appear as blurry dots in MFI and show very limited morphological attributes in FlowCAM (Fig. 2 and Fig. 3, respectively).

Another interesting finding from our study is that the FIM techniques were able to detect clustered or aggregated cells. It may be expected that clustered cells will always be not picked up with the conventional techniques, given the low concentration of this population (Fig. 5) and low sample volume measured by these techniques. It should be noted that one cannot exclude that some of the aggregates detected by FIM are artefacts created by the transition of the wide tubing diameter to the narrow flow cell, which condenses the cells in response to spatial shrinking. However, the aggregates were also detected when more diluted samples (up to 16×) were measured with FIM techniques. Furthermore it is shown that aggregates are formed around decaying and dead cells (26).

Analysis of the FIM parameters revealed clear changes during storage in the majority of the parameters highlighted in this study, namely ECD, intensity mean, intensity SD and aspect ratio for MFI; and ABD, symmetry, aspect ratio, and circle fit for FlowCAM. Both MFI and FlowCAM revealed a decreasing trend in the size (ECD and ABD, respectively) of the cells during the storage for up to eight days under ambient conditions. Moreover, cells at later time points (as of day 1) appeared to have a higher intensity, a lower intensity SD and smaller aspect ratios (see Supplementary Fig. S2 and S3). The latter, together with lower symmetry and circle fit values, indicate that the cells became less symmetric and more elongated in shape. All these observations point towards a decrease in the population of live cells and/or changes in the quality of the live cell population, e.g., because of apoptosis. Shrinkage of cell size and changes in cell shape are observed for dead and dying cells whose concentrations are expected to increase under stress (14). Furthermore, a decrease in the intensity SD can be a sign of disappearance of the cell organelles that contributed to variations in intensity of the image of a cell. Further investigation revealed that upon storage at ambient temperature also within the defined populations (e.g., live and dead cells) changes in morphology were observed, most obviously in the FlowCAM data. This suggests that in particular FlowCAM is able to pick up early stages of loss in viability that may cause changes in the transparency and shape of the cells (14).

It has to be noted that the two FIM techniques described herein offer several other morphological parameters that could be used in analysis of the cells. However, the combination of a high-magnification lens and a thin focus plane of the flow cell results in substantial numbers of imaged particles that were out of focus. These particles affect the values of a few morphological parameters (e.g., intensity), and therefore were not included in our study.

During the development of our FIM based methods, we have used one type of cells, i.e., B-ALL cell lines. However, CBMPs may contain cells with different morphological properties than B-ALL cells or contain multiple cell types (27). In principle, one can apply the same approach to other cell types and heterogeneous cell populations. Therefore, one needs to develop specific software filters for each type of cells.

Our investigation shows that FIM techniques have the potential to determine total cell concentration and cell viability. This provides a strong basis for more detailed and specific studies in order to validate FIM-based methods for a broad range of CBMPs. The FIM techniques, just like the conventional techniques, were able to detect differences in cell viability between the two cell lines. This may be caused by the difference in genetic background or disease status of the donors. Moreover, we have shown other potentials that these techniques can offer in characterization of CBMPs. The capability of detecting and imaging cell debris, cell aggregates and potentially different cell types offers an excellent application in characterization of impurities in CBMPs.

## Conclusion

In this study we have developed label-free methods for cell concentration and viability determination based on two different FIM techniques, MFI and FlowCAM. Our data suggests that both methods deliver fairly similar results for total cell concentration and cell viability as traditional methods, i.e., hemocytometry and the automated cell counting. Whereas the MFI based method showed a higher precision with respect to determination of the total cell concentration, the FlowCAM based method provides higher-resolution images. The latter may be useful to identify non-cellular particles and potentially discriminate between different types of cells.

## Electronic supplementary material

ESM 1 (DOCX 0.99 MB)

## Abbreviations

ABD: Area based diameter
B-ALL: B-lineage acute lymphoblastic leukemia
CBMPs: Cell-based medicinal products
DMSO: Dimethylsulfoxide
K_2_HPO_4_: Dipotassium phosphate
ECD: Equivalent circular diameter
FACS: Fluorescence-assisted cell sorting
FIM: Flow imaging microscopy
FSC: Forward scatter
HSA: Human serum albumin
MFI: Micro-flow imaging
MVAS: MFI View analysis suite
MVSS: MFI View system software
NaCl: Sodium chloride
SD: Standard deviation
SSC: Side scatter
