# Supplementary material for: Label-Free, Flow-Imaging Methods for Determination of Cell Concentration and Viability
Source: Pharm Res. 2018 May 30;35(8):150. doi: 10.1007/s11095-018-2422-5 (PMC5976703; doi:10.1007/s11095-018-2422-5)
Supplement: Supplementary file 1 — (DOCX 0.99 MB) [file 11095_2018_2422_MOESM1_ESM.docx]

**Supplementary Information**

**Label-free, flow-imaging methods for determination of cell concentration and viability**

A.S. Sediq^1^, R. Klem^1^, M.R. Nejadnik^1^, P. Meij^2^, W. Jiskoot^1,^*

^1^ Division of BioTherapeutics, Leiden Academic Centre for Drug Research (LACDR), Leiden University, Leiden, The Netherlands

^2^ Department of Clinical Pharmacy and Toxicology, Leiden University Medical Center, Leiden, The Netherlands

* Corresponding author:

Wim Jiskoot

Email: [w.jiskoot@lacdr.leidenuniv.nl](mailto:w.jiskoot@lacdr.leidenuniv.nl)

Phone: +31 71 527 4314;

Fax: +31 71 527 4565


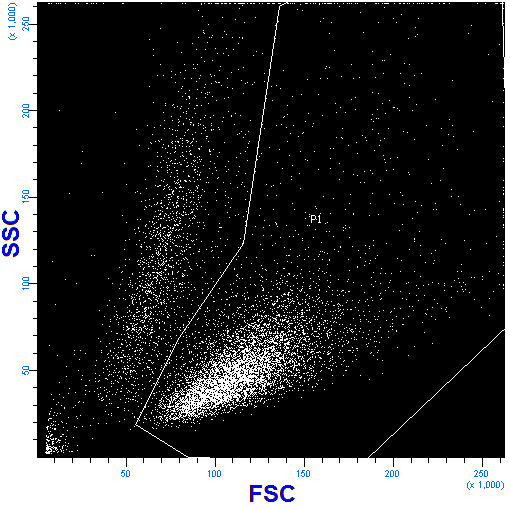


**Supplementary Figure S1**: Gating strategy for the FACS supported sorting of dead/dying and viable cells. The graph shows the FSC – SSC plot for the sample containing fresh cells from cell line 1. The gated population is considered viable.

**Supplementary Figure S2:** Overview of different morphological parameters of all the particles counted with MFI in samples of cell line 1 during 8 days of storage. For each parameter the frequency distribution of the corresponding parameter unit range is shown.

**Supplementary Figure S3:** Overview of different morphological parameters of all the particles counted with MFI in samples of cell line 2 during 8 days of storage. For each parameter the frequency distribution of the corresponding parameter unit range is shown.

**Supplementary Figure S4:** Overview of different morphological parameters of all the particles counted with FlowCAM in samples of cell line 1 during 8 days of storage. For each parameter the frequency distribution of the corresponding parameter unit range is shown

**Supplementary Figure S5:** Overview of different morphological parameters of all the particles counted with FlowCAM in samples of cell line 2 during 8 days of storage. For each parameter the frequency distribution of the corresponding parameter unit range is shown.
